# Hsa-miR-499 rs3746444 Polymorphism Contributes to Cancer Risk: A Meta-Analysis of 12 Studies

**DOI:** 10.1371/journal.pone.0050887

**Published:** 2012-12-07

**Authors:** Man-Tang Qiu, Jing-Wen Hu, Xiang-Xiang Ding, Xin Yang, Zhi Zhang, Rong Yin, Lin Xu

**Affiliations:** 1 The Fourth Clinical College of Nanjing Medical University, Nanjing, China; 2 Department of Thoracic Surgery, Nanjing Medical University Affiliated Cancer Hospital Cancer Institute of Jiangsu Province, Nanjing, China; 3 The First Clinical College of Nanjing Medical University, Nanjing, China; Kinghorn Cancer Centre, Garvan Institute of Medical Research, Australia

## Abstract

**Background:**

Single nucleotide polymorphisms (SNPs) occurred in pre-microRNAs or targets of microRNAs (miRs) may contribute to cancer risks. Since 2007, many studies have investigated the association between common SNPs located on hsa-miR-499 (rs3746444) and cancer risks; however, the results were inconclusive.

**Methodology/Principal Findings:**

We conducted a meta-analysis of 12 studies that included 5765 cases and 7076 controls to identify the strength of association. Odds ratio (OR) and 95% confidence intervals (95% CI) were used to assess the strength of association. Overall, individuals with the variant AG (OR = 1.215, 95% CI: 1.027, 1.437; P_heterogeneity_<0.01) and AG/GG (OR = 1.227, 95% CI: 1.046, 1.439; P_heterogeneity_<0.01) genotypes were associated with a significantly increased risk of cancer than those with wild AA genotype. Sub-group analysis revealed that the variant AG (OR = 1.411, 95% CI: 1.142, 1.745; P_heterogeneity_ = 0.01) and AG/GG (OR = 1.413, 95% CI: 1.163, 1.717, P_heterogeneity_ = 0.01) genotypes still showed an increased risk of cancer in Asians; however, a trend of reduced risk of cancer was observed in Caucasians (AG vs. AA: OR = 0.948, 955 CI: 0.851, 1.057, P_heterogeneity_ = 0.12; AG/GG vs. AA: OR = 0.959, 95% CI: 0.865, 1.064; P_heterogeneity_ = 0.19). Meta-regression showed that ethnicity (p = 0.048) and sample size (p = 0.02) but not cancer types (p = 0.89) or source of control (p = 0.97) were the sources of heterogeneity.

**Conclusions:**

These meta-analysis results suggest that hsa-miR-499 polymorphism rs3746444 is associated with a significantly increased risk of cancer, especially in Asian populations.

## Introduction

MicroRNAs (miRNAs) are a kind of non-coding RNAs, about ∼22 nucleotides in length. Mature miRNAs target the 3′ untranslated region of mRNA, leading to mRNA degradation or suppression of translation [1], [2]. It is reported that a single miRNA could bind to mRNAs of about 200 genes, therefore miRNAs play an important role in gene regulation [3], [4] and are involved in physiologic and pathologic processes [1], including tumorigenesis [5], proliferation [6], apoptosis [7], and metabolism [8].

MiRNA-499 plays an important role in tumor biology and is associated with progression [9] and prognosis of cancer [10]. In 2010, Hu and colleagues [10] reported that miRNAs expression levels in serum altered greatly and the miRNA-499 level was a prognostic factor of patients with non-small cell lung cancer.

Common single nucleotide polymorphisms (SNPs) in pre-miRNAs and cancer risk has been investigated by case-control studies in the last decade, and some common SNPs in pre-miRNAs have been demonstrated with an increased cancer risk, such as hsa-miR-196a2 rs11614913 [11], [12] and hsa-miR-146a rs2910164 [11], [13] polymorphisms. Another common SNP in pre-miRNA, rs3746444 in hsa-miR-499 (A>G), was also studied in several kinds of cancer, such as breast cancer [14]–[16], liver cancer [17], cervical squamous cell cancer [18], and gastric cancer [19]. However, these studies yielded different or even controversial results. For example, Hu found that the G variant allele carriers had an increased risk of breast cancer [16], but Catucci reported no significant association [15]; Liu found that the AG and AG/GG genotype were associated with a reduced risk of squamous cell cancer of head and neck [20], however, Chu and colleagues found an increased risk of oral squamous cancer [21].

To confirm the association between hsa-miR-499 rs3746444 polymorphism and cancer risk, we performed this meta-analysis by pooling all eligible studies to calculate the estimate of overall cancer risk and evaluated influence of cancer types and ethnicity.

## Methods

### Identification of eligible studies

Eligible case-control studies were extracted by electronic search of databases and manual search of references of relative articles and reviews. In order to identify as many relative articles as possible, PubMed and China National Knowledge Infrastructure (CNKI) were searched using key words “microRNA”, “polymorphism”, and “cancer”. There was no limitation of research and the last research was performed on August 8, 2012. References of related studies and reviews were manually searched for additional studies.

### Inclusion and exclusion criteria

Studies were selected according to the following inclusion criteria: (1) case-control studies; (2) investigating the association between miR-499 3746444 (A>G) SNP and cancer risks; (3) cancers diagnosed by histopathology; (4) providing detail genotype frequencies. Studies without detail genotype frequencies were excluded. Titles and abstracts of searching results were screened and full text papers were further evaluated to confirm eligibility. Two reviewers (Qiu and Hu) extracted eligible studies independently according to the inclusion criteria. Disagreement between two reviewers was discussed with another reviewer (Yang) till consensus was achieved.

### Data extraction

Data of eligible studies was extracted by two reviewers (Qiu and Hu) independently in duplicate with a standard data-collection form. The following data was collected: name of first author, year of publication, country where the study was conducted, genotyping methods, ethnicity, cancer types, source of control, Hardy-Winberg equilibrium, number of cases and controls, genotype frequency in cases and controls. Different ethnicity descents were categorized as Asian and Caucasian. Cancer types were classified as breast cancer, liver cancer (hepatocellular carcinoma and liver cancer), squamous cancer (squamous cell carcinoma of head and neck, cervical squamous cell carcinoma, and oral squamous cell carcinoma), and other cancers (gastric cancer and bladder cancer). Eligible studies were defined as hospital-based (HB) and population-based (PB) according to the control source. When Hardy-Winberg equilibrium (HWE) in the controls was not reported, an online program (http://ihg.gsf.de/cgi-bin/hw/hwa1.pl) was used to test the HWE by chi-square test for goodness of fit [22]. Two reviewers reached consensus on each item.

### Methodological quality assessment

The quality of eligible studies was evaluated by three reviewers (Qiu, Hu, and Yang) independently by scoring according to a “methodological quality assessment scale” (see supplemental information “Table S2: Scale for methodological quality assessment”), which was modified form a previous meta-analysis [23]. In the scale, 6 items were assessed, namely the representativeness of cases, source of controls, ascertainment of relevant cancer, sample size, quality control of genotyping methods, and Hardy-Weinberg equilibrium (HWE). Quality scores ranged from 0 to 10 and a high score indicated good quality of the study. Three reviewers solved disagreement by discussion.

### Statistical analysis

The association strength between has-miR-499 rs3746444 (A>G) polymorphism and cancer risks was measured by odds ratio (OR) with 95% confidence intervals (95% CI). The estimates of pooled ORs were achieved by calculating a weighted average of OR from each study. A 95% CI was used for statistical significance test and a 95% CI without 1 for OR indicating a significant increased or reduced cancer risk. The pooled ORs were calculated for homozygote comparison (GG versus AA), heterozygote comparison (AG versus AA), dominant (AG/GG versus AA) and recessive (GG versus AG/AA) modes, assuming dominant and recessive effects of the variant G allele, respectively. Subgroup analyses were also conducted to explore the effects of confounding factors: cancer types, ethnicities, and source of control. Sensitivity analyses were performed to indentify individual study' effect on pooled results and test the reliability of results.

Chi-square based Q test was used to check the statistical heterogeneity between studies, and the heterogeneity was considered significant when p<0.10 [24]. The fixed-effects model (based on Mantel-Haenszel method) and random-effects model (based on DerSimonian-Laird method) were used to pool the data from different studies. The fixed-effects model was used when there was no significant heterogeneity; otherwise, the random-effects model was applied [25]. Meta-regression was performed to detect the source of heterogeneity. The between studies variance (τ^2^) was used to quantify the degree of heterogeneity between studies and the percentage ofτ^2^ was used to describe the extent of heterogeneity explained [26].

Publication bias was detected with Begg's funnel plot and the Egger' linear regression test, and a p<0.05 was considered significant [27]. All statistical analyses were calculated with STATA software (version 10.0; StataCorp, College Station, Texas USA). And all P values were two-side.

## Results

### Characteristics of eligible studies

**Figure 1:**
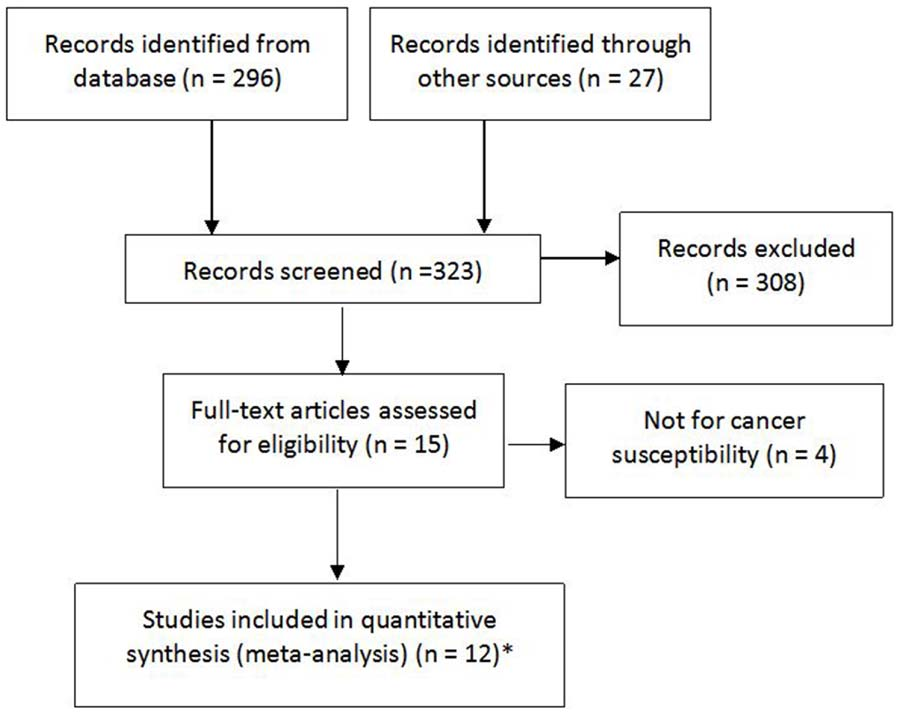
PRISMA Flow Chart. *a total of 11 articles were identified and two separate studies were reported in one articles, thus 12 studies were eligible.

In total, 11 articles [14]–[21], [28]–[30] were identified according to inclusion and exclusion criteria. The detailed screening process was shown in Figure 1. After reviewing full text articles, 4 studies [10], [31]–[33] were excluded for the reason of not for cancer susceptibility. In the study reported by Catucci and colleagues [15], participants were recruited from German and Italy and the genotype frequencies were presented separately, thus each of them was considered as a separate study in this meta-analysis. Therefore, a total of 12 case-control studies, including 5765 cancer cases and 7076 controls, assessing the association between has-miR-499 rs3746444 polymorphism and cancer risk were included. Among the 12 eligible studies, 4 of them were studies of Caucasian [15], [20], [28] and 8 studies were of Asian [14], [16]–[19], [21], [29], [30] (details shown in Table 1). Cancer cases were diagnosed histologically or pathologically in all studies. Polymerase chain reaction-restriction fragment length polymorphism (PCR-RFLP) assay was used for genotyping in 10 studies [16]–[21], [28]–[30] and TaqMan genotyping assay was performed in the other 2 studies [14], [15]. Blood sample was used for genotyping in all studies. Genotyping assay quality control was performed in 7 studies [14]–[17], [20], [21]. HWE of genotype distribution in the controls was tested in 8 studies [16]–[18], [20], [21], [28]–[30] and they were all in consistent with HWE. In the 4 studies [14], [15], [19] which did not reported HWE, the online program was used to test HWE in controls and only one study reported by Okubo [19] was not in agreement with HWE (p = 0.048).

**Table 1:** Characteristics of Eligible Studies.

First Author	Year	Country	Ethnicity	Cancer Type	Control	Cases			Controls		
						AA	AG	GG	AA	AG	GG
Hu [16]	2009	China	Asian	BC	PB	707	258	44	816	248	29
Catucci [15]	2010	Germany	Caucasian	BC	PB	536	250	37	601	290	34
Catucci [15]	2010	Italy	Caucasian	BC	PB	414	295	47	704	452	86
Liu [20]	2010	USA	Caucasian	SCCHN	HB	745	309	55	710	366	54
Okubo [19]	2010	Japan	Asian	GC	HB	364	151	37	466	198	33
Akkız [28]	2011	Turkey	Caucasian	HCC	HB	45	87	90	47	93	82
Mittal [29]	2011	India	Asian	BLC	HB	95	92	25	121	94	35
Zhou [18]	2011	China	Asian	CSCC	HB	134	84	8	223	71	15
Chu [21]	2012	China	Asian	OSCC	HB	339	119	12	356	66	3
Zhou [17]	2012	China	Asian	LC	HB	141	41	4	371	100	12
Xiang [30]	2012	China	Asian	HCC	HB	36	40	24	106	71	23
Alshatwi [14]	2012	Saudi Arabia	Asian	BC	PB	30	62	8	45	40	15

BC: breast cancer; SCCHN: squamous cell carcinoma of head and neck; GC: gastric cancer; HCC: hepatocellular carcinoma; BLC: bladder cancer; CSCC: cervical squamous cell carcinoma; OSCC: oral squamous cell carcinoma; LC: liver cancer; PB: population-based; HB: hospital-based.

### Meta-analysis results

**Figure 2:**
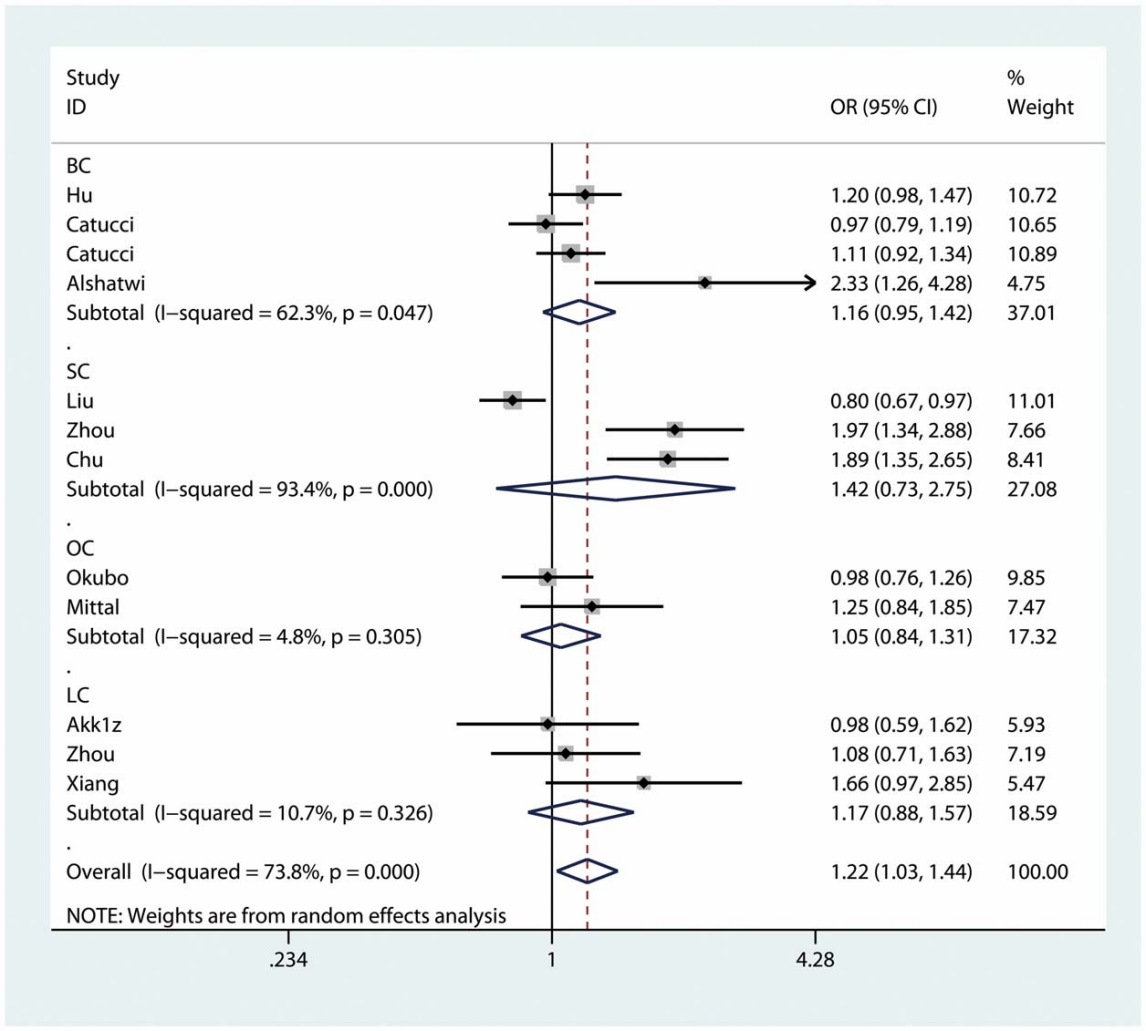
Forest plot of heterozygote comparison for overall comparison (AG vs. AA). BC: breast cancer; SC: squamous cancer; OC: other cancers; LC: liver cancer.

**Figure 3:**
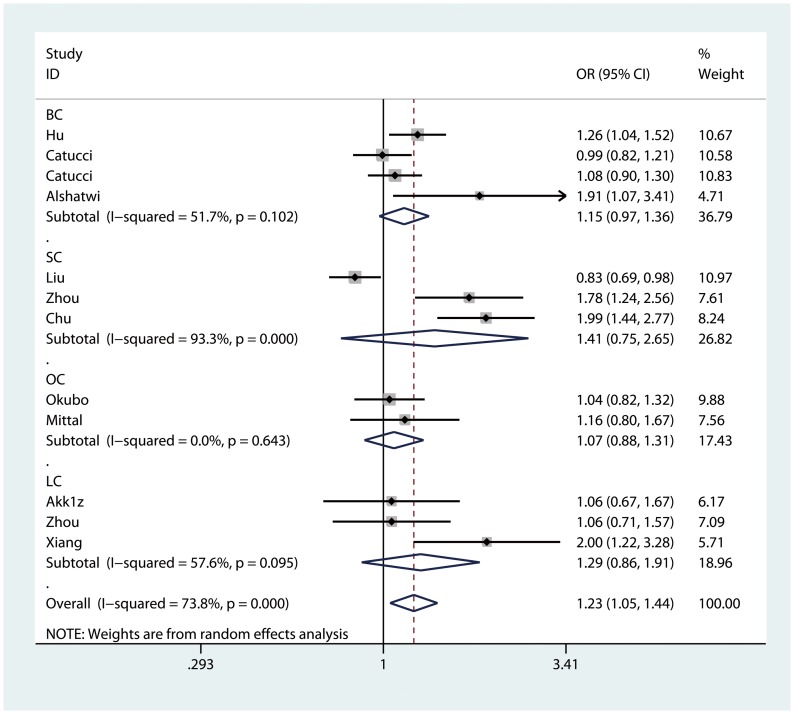
Forest plot of dominant model for overall comparison (AG/GG vs. AA). BC: breast cancer; SC: squamous cancer; OC: other cancers; LC: liver cancer.

We observed a significantly increased risk of cancer susceptibility in heterozygote comparison (AG vs. AA: OR = 1.215, 95% CI: 1.027, 1.437; P_heterogeneity_<0.01, Figure 2) and dominant model (AG/GG vs. AA: OR = 1.227, 95% CI: 1.046, 1.439; P_heterogeneity_<0.01, Figure 3) when all eligible studies were pooled. The association strength between hsa-miR-499 rs3746444 polymorphism and cancer risk was shown in Table 2. As shown in Table 2, no significant association was found in homozygote comparison (GG vs. AA: OR = 1.236, 95% CI: 0.988, 1.546; P_heterogeneity_ = 0.06) or recessive model (GG vs. AG/AA: OR = 1.164, 95% CI: 0.935, 1.449; P_heterogeneity_ = 0.04), however, a trend of increased risk could be drawn.

**Table 2:** Meta-analysis Results.

		GG vs AA		AG vs AA		GG/AG vs AA		GG vs AA/AG	
	N	OR	P_h_	OR	P_h_	OR	P_h_	OR	P_h_
Total	12	1.236(0.988,1.546)	0.06	1.215(1.027,1.437)*	<0.001	1.227(1.046,1.439)*	<0.001	1.164(0.935,1.449)	0.043
Cancer Types									
BC	4	1.165(0.915,1.482)	0.189	1.163(0.952,1.420)	0.047	1.128(1.013,1.256)*	0.102	1.065(0.712,1.595)	0.059
LC	3	1.531(0.727,3.226)	0.047	1.170(0.890,1.538)	0.326	1.285(0.864,1.913)	0.095	1.355(0.991,1.852)	0.106
SC	3	1.276(0.620,2.627)	0.087	1.421(0.735,2.749)	<0.001	1.412(0.751,2.653)	<0.001	1.103(0.791,1.539)	0.107
others	2	1.186(0.818,1.720)	0.238	1.048(0.848,1.296)	0.305	1.074(0.880,1.310)	0.643	1.127(0.786,1.616)	0.13
Ethnicities									
Asian	8	1.430(0.996,2.051)	0.056	1.411(1.142,1.745)*	0.006	1.413(1.163,1.717)*	0.009	1.241(0.841,1.833)	0.018
Caucasian	4	1.031(0.833,1.276)	0.795	0.948(0.851,1.057)	0.124	0.959(0.865,1.064)	0.188	1.055(0.866,1.285)	0.685
Source of Control									
PB	4	1.165(0.915,1.482)	0.189	1.163(0.952,1.420)	0.047	1.128(1.013,1.256)*	0.102	1.065(0.712,1.595)	0.059
HB	8	1.295(0.935,1.793)	0.048	1.239(0.951,1.615)	<0.001	1.269(0.982,1.641)	<0.001	1.222(0.922,1.620)	0.089
Number of participants									
Larg^a^	5	1.168(0.963,1.416)	0.219	1.001(0.867,1.156)	0.042	1.028(0.891,1.185)	0.029	1.182(0.944,1.479)	0.248
Smal^b^	7	1.304(0.837,2.033)	0.042	1.512(1.196,1.912)*	0.062	1.494(1.187,1.881)*	0.044	1.117(0.725,1.720)	0.022

N: number of studies included; OR: odds ratio; P_h_: p value for heterogeneity; BC: breast cancer; LC: liver cancer; SC: squamous cancer; PB: population-based; HB: hospital-based;

* OR with statistical significance; a: studies with more than 1000 participants; b: studies with less than 1000 participants.

We then performed sub-group analyses to investigate the effect of cancer types, ethnicity, and source of control. As for cancer types, increased cancer risk was only found in the dominant model comparison for breast cancer (AG/GG vs AA: OR = 1.128, 95% CI: 1.013, 1.256; P_heterogeneity_ = 0.102). In the sub-group analyses of “liver cancer”, “squamous cancer”, and “other cancers”, we did found any significant association between hsa-miR-499 rs3746444 polymorphism and cancer risk. In a coincidence, the 4 studies of breast cancer were all population-based, thus an increased risk was found in dominant model comparison. As for hospital-based studies, we did not found any significant association between miR-499 polymorphism and cancer risk.

Ethnicity, however, affected cancer susceptibility greatly. In Asians, there was a statistically increased cancer risk in the comparison of heterozygote (AG vs. AA: OR = 1.411, 95% CI: 1.142, 1.745; P_heterogeneity_ = 0.01) and dominant model (AG/GG vs. AA: OR = 1.413, 95% CI: 1.163, 1.717, P_heterogeneity_ = 0.01). The results in Asians were similar to that of overall comparisons of pooled eligible studies. In Caucasians, however, no significant association was found in each comparison. On the other hand, a trend of reduced cancer risk could be drawn from heterozygote comparison (AG vs. AA: OR = 0.948, 955 CI: 0.851, 1.057; P_heterogeneity_ = 0.12) and dominant model (AG/GG vs. AA: OR = 0.959, 95% CI: 0.865, 1.064; P_heterogeneity_ = 0.19). Taken together, these results revealed that hsa-miR-499 rs3746444 polymorphism was only associated with an increased risk of cancer in Asians.

### Heterogeneity

Heterogeneity between studies in each comparison was shown in Table 2. We investigated the source of heterogeneity by cancer types, source of control, ethnicity, and sample size (studies with more than 1000 participants were categorized as “large”, and studies with less 1000 participants were categorized as “small”) with meta-regression in variant heterozygote comparison (AG vs. AA). Meta-regression results revealed that ethnicity (p = 0.05) and sample size (p = 0.02) but not cancer types (p = 0.89) or source of control (p = 0.97) contributed to the source of heterogeneity. Additionally, ethnicity could explain 34.21% of the between studies variance (τ^2^), and sample size could explain 53.44% of the variance (τ^2^).

### Sensitivity analysis

Sensitivity analysis was performed to explore individual study's influence on the pooled results by deleting one single study each time from pooled analysis. The results showed that no individual study affected the pooled OR significantly, since no substantial change was found (figure not shown).

### Publication bias

**Figure 4:**
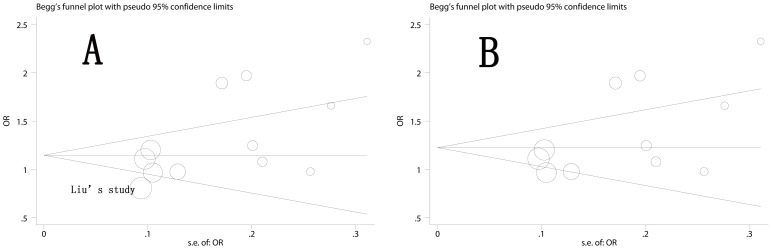
Funnel plot of heterozygote comparison (AG/GG vs. AA). A: funnel plot of all 12 eligible studies, Egger's test p = 0.02; B: funnel plot of 11 studies (Liu's study was excluded), Egger's test p = 0.06; the circles represent the weight of individual study.

Publication bias was assessed by Begg's funnel plot and Egger's test. Begg's funnel plot was roughly symmetrical (p = 0.15 for AG versus AA) (Figure 4.A). Egger's test was then performed for statistical test and publication bias was detected (p = 0.02 for AG versus AA). Further study revealed that the study reported by Liu and colleagues [20] was responsible for the asymmetry of funnel plot (Figure 4.A). When this study was deleted, there was no evidence of publication bias (p = 0.06 for AG vs AA, Figure 4.B), and the pooled OR was still significant (OR = 1.268, 95% CI: 1.081, 1.488), and the between studies variance (τ^2^) also decreased from 0.06 to 0.04.

## Discussion

In this meta-analysis, 12 eligible studies [14]–[21], [28]–[30], including 5765 cancer cases and 7076 controls, were identified and analyzed. We demonstrated that hsa-miR-499 rs3746444 polymorphism was associated with a statistically increased risk of cancer in the variant AG heterozygote and AG/GG genotype compared with the AA wild-type homozygote. This association was significant in Asians, however, an opposite trend was found in Caucasians.

It is believed that a SNP in the pre-miRNAs could influence the processing and binding property of mature miRNAs [34], [35]. Together with the critical role of miRNAs in gene regulation, the variations in miRNAs would be related to cancer risks [16], [23], [35]. In 2011, Liu and colleagues [9] found that miRNA-499-5p could promote cellular invasion and tumor metastasis in colorectal cancer by targeting FOXO4 and PDCD4 and miRNA-499-3p (3746444 A>G) was found in an invasive breast cancer cell line [34]. Hu also found miRNA 499 expression level in serum was a prognostic factor in NSCLC [10]. Given the important role of miRNA 499, it is reasonable that rs3746444 (A>G) may contribute to cancer susceptibility.

Among 12 eligible studies [14]–[21], [28]–[30], G allele variant carriers were reported with an increased risk of breast cancer [16], cervical squamous cell cancer [18], oral squamous cell cancer [21], and hepatocellular carcinoma [30], and the significant association was mostly found in the heterozygote comparison (AG vs. AA) and dominant model (AG/GG vs. AA), which was in consistent with our pooled analysis. Liu and colleagues [20] also found a significantly reduced cancer risk with AG and AG/GG genotypes of hsa-miR-499. These results suggested that the variant AG and AG/GG genotypes of hsa-miR-499 were definitive associated with cancer susceptibility.

In the sub-group analysis of cancer types, no significant association was found except for dominant model comparison of breast cancer. But for the 4 studies of breast cancer [14]–[16], 2 of them found increased risk with G variant allele carriers [14], [16]. In addition, in the sub-group of squamous cancer, there was no significant association either, although all of the 3 individual studies reported increased [18], [21] or reduced [20] cancer risk with miR-499 polymorphism. This discrepancy may be explained by the reason that the sample size of the studies was relatively small and there was a high possibility of chance due to insufficient statistical power. Additionally, ethnicity was also an important reason, because the studies reported increased risk were carried out in Asians.

During sub-group analyses, we found that ethnicity greatly affected the association between hsa-miR-499 rs3746444 polymorphism and cancer risk. As mentioned in the part of result, there was an increased cancer risk of AG and AG/GG genotype in Asians, but a trend of reduced cancer risk was found in Caucasians. The different cancer risks in Asians and Caucasians was also reported in other meta-analyses [11], [12], [22], [23]. The differences may be explained by genetic diversities, different risk factors in life styles, and the exposure to different environmental factors.

As for the aforementioned publication bias detected (AG vs. AA) by Egger' test, Liu' study [20] was responsible for the bias. However, Liu's study [20] was the only study which reported reduced cancer risk with squamous cell carcinoma of head and neck in Caucasians. Additionally, we also observed a tendency of reduced risk of hsa-miR-499 rs3746444 polymorphism in Caucasians. Thus, we speculated that the publication bias we detected was not a favor to publish positive results, but the fact that current studies conducted in Caucasians were too few. It is expected that when more studies in Caucasians are published, the funnel plot will be more symmetrical and no publication bias will be detected.

For heterogeneity, we found ethnicity and sample size were the source of heterogeneity. Although studies of small size may contribute to a small-study effect, in which effects reported are larger, and lead to between studies variance, sample size was not considered for heterogeneity in previous meta-analyses. However, this kind heterogeneity is difficult to exclude, because recruitment of enough cases with specific kind of cancer is difficult.

In this meta-analysis, we included 5765 cancer cases and 7076 controls, which can provide enough statistical power and strengthened the reliability of our results. Upon including eligible studies, a methodological quality assessment was conducted and all studies had acceptable quality. In addition, there was no limitation of languages when searching, thus there was a low chance of selection bias. Some limitation of our meta-analysis should be considered. Firstly, individual data was not available and a more precise adjusted OR for other covariates such as age, family history, and environment factors was not allowed. Secondly, the number of studies included for sub-group analysis of cancer types was too small.

In conclusion, we demonstrate that hsa-miR-499 rs3746444 polymorphism is associated increased cancer risk, especially in Asians. To confirm this association, future large size case-control studies are required, especially in Caucasians.

## Supporting Information

Table S1 PRISMA checklist.
(DOC)

Table S2 Scale for methodological quality assessment.
(DOC)
