# Supplementary material for: Hsa-miR-499 rs3746444 Polymorphism Contributes to Cancer Risk: A Meta-Analysis of 12 Studies
Source: PLoS One. 2012 Dec 7;7(12):e50887. doi: 10.1371/journal.pone.0050887 (PMC3517592; doi:10.1371/journal.pone.0050887)
Supplement: Table S2 — Scale for methodological quality assessment. (DOC) [file pone.0050887.s002.doc]

| **Table S2.** **Scale for methodological quality assessment.** | |
| --- | --- |
| Criteria | Score |
| 1.Representativeness of cases |  |
| Selected from cancer registry or multiple cancer center sites | 2 |
| Selected from oncology department or cancer institute | 1 |
| Not described | 0 |
| 2.Source of controls |  |
| Population or community based | 2 |
| Hospital-based cancer-free controls | 1.5 |
| Healthy volunteers without total description | 1 |
| Cancer-free controls with related diseases | 0.5 |
| Not described | 0 |
| 3.Ascertainment of relevant cancer |  |
| Histopathologic confirmation | 2 |
| Patient medical record | 1 |
| Not described | 0 |
| 4.Sample size |  |
| >1000 | 2 |
| 200-1000 | 1 |
| <200 | 0 |
| 5.Quality control of genotyping methods |  |
| Repetition of partial/total tested samples with a different method | 1 |
| Repetition of partial/total tested samples with the same method | 0.5 |
| Not described | 0 |
| 6.Hardy-Weinberg equilibrium (HWE) |  |
| Hardy-Weinberg equilibrium in control subjects | 1 |
| Hardy-Weinberg disequilibrium in control subjects | 0 |
